# Geographical variation in the progression of type 2 diabetes in Peru: The CRONICAS Cohort Study

**DOI:** 10.1016/j.diabres.2016.09.007

**Published:** 2016-11

**Authors:** Antonio Bernabé-Ortiz, Rodrigo M. Carrillo-Larco, Robert H. Gilman, Catherine H. Miele, William Checkley, Jonathan C. Wells, Liam Smeeth, J. Jaime Miranda, CRONICAS Cohort Study Group

**Affiliations:** a CRONICAS Center of Excellence in Chronic Diseases, Universidad Peruana Cayetano Heredia, Lima, Peru; b Faculty of Epidemiology and Population Health, London School of Hygiene and Tropical Medicine, London, United Kingdom; c Program in Global Disease Epidemiology and Control, Department of International Health, Bloomberg School of Public Health, Johns Hopkins University, Baltimore, USA; d Biomedical Research Unit, Asociación Benéfica PRISMA, Lima, Peru; e Division of Pulmonary and Critical Care, School of Medicine, Johns Hopkins University, Baltimore, USA; f Childhood Nutrition Research Centre, UCL Institute of Child Health, University College London, London, United Kingdom; g Department of Medicine, School of Medicine, Universidad Peruana Cayetano Heredia, Lima, Peru

**Keywords:** Type 2 diabetes, Incidence, Altitude, Risk factors, Obesity

## Abstract

**Background:**

The study aims were to estimate the incidence and risk factors for T2D in four settings with different degree of urbanization and altitude in Peru.

**Methods:**

Prospective cohort study conducted in urban, semi-urban, and rural areas in Peru. An age- and sex-stratified random sample of participants was taken from the most updated census. T2D was defined as fasting blood glucose ⩾7.0 mmol/L or taking anti-diabetes medication. Exposures were divided into two groups: geographical variables (urbanization and altitude), and modifiable risk factors. Incidence, relative risks (RR), 95% confidence intervals (95%CI), and population attributable fractions (PAF) were estimated.

**Results:**

Data from 3135 participants, 48.8% males, mean age 55.6 years, was analyzed. Overall baseline prevalence of T2D was 7.1% (95%CI 6.2–8.0%). At follow-up, including 6207 person-years of follow-up, a total of 121 new T2D cases were accrued, equating to an incidence of 1.95 (95%CI 1.63–2.33) per 100 person-years. There was no urban to rural gradient in the T2D incidence; however, compared to sea level sites, participants living in high altitude had a higher incidence of diabetes (RR = 1.58; 95%CI 1.01–2.48). Obesity had the highest attributable risk for developing T2D, although results varied by setting, ranging from 14% to 80% depending on urbanization and altitude.

**Conclusions:**

Our results suggest that the incidence of T2D was greater in high altitude sites. New cases of diabetes were largely attributed to obesity, but with substantial variation in the contribution of obesity depending on the environment. These findings can inform appropriate context-specific strategies to reduce the incidence of diabetes.

## Highlights

• The incidence of type 2 diabetes in the Latin American region is poorly defined.
• There was no evidence that urbanization altered the incidence of diabetes.
• High altitude sites were found to have a higher risk of developing diabetes.
• Obesity, varying across settings, had the highest attributable risk for diabetes.
• This study improves the understanding of diabetes burden and identifies hotspots.

## 1 Introduction

The burden of type 2 diabetes (T2D) is rising worldwide: according to estimates from 2014, about 347 million people are living with T2D [1]. Although T2D is preventable and controllable through lifestyle modification, weight reduction, and medication use [2], a recent systematic analysis reported an increasing trend in the worldwide prevalence of T2D: from 8.3% among men and 7.5% among women in 1980, to 9.8% and 9.2% in men and women in 2008 [3].

Information regarding the impact of risk factors on the development of T2D in low- and middle-income countries (LMIC) comes from cross-sectional reports instead of longitudinal studies [4]. The heterogeneity of T2D and its dependence on environmental factors support the need for population-based, ethnically focused, and country-specific studies of T2D incidence [5]. Given that much of the available literature about the incidence of T2D arises from high-income countries, no direct extrapolation can be made for ethnic minorities and other populations living in and interacting with different environments. These populations matter because they may have lesser or poorer access to information or healthcare services, including diagnosis and treatment. Beyond the health sector, these populations present also different lifestyle traits that are largely driven by the environment they interact with, including a wide range of social disparities across the lifespan. Taken together, these macro forces place them at much higher risk of impaired T2D-related health outcomes including micro-vascular or macro-vascular complications and mortality. As economical but also human resources are scarce in LMIC, such information would provide a much needed strong platform to implement appropriate strategies to tackle the progression of T2D in LMIC contexts [6].

Peru is a middle-income country where noncommunicable diseases are responsible for more than half of all causes of deaths and 42% of total years of life lost [7]. Its diverse geography—ranging from sea level to Andean mountains to jungle rainforest areas—alongside unequal societal development, and the rapid epidemiological and nutrition transition undergoing in the country [8], provides a unique scenario where urban, semiurban, and rural settings coexist. Many LMIC-based studies oriented to characterize the burden of noncommunicable diseases have largely focused on urban and rural differentials [9]. Given the complex human-environment interactions, and considering the physiological responses introduced, for example adapting to high altitude areas [10], very little is known about the effect of introducing combinations of rural and urban settings together with lowland and high altitude environments on shaping the burden of T2D. So far, available cross-sectional reports signal towards a link between living at high altitude and lower prevalence of T2D [11], [12], but they cannot determine causality. Longitudinal studies looking at the geographical variation on the progression towards T2D by altitude settings are scarce or even absent in LMIC. Given the large population living at high altitude around the globe [13], studying the incidence of T2D in high altitude settings warrants attention.

Peru’s geographical predominance in the Andean region makes it well positioned to adequately explore to what extent altitude may play a role in T2D heterogeneity in the velocity of transitioning towards metabolic disorders, including diabetes. As a result, there is an opportunity to better understand the effects of rapid urbanization and geographical variation on the progression of noncommunicable diseases, especially focusing on T2D. Likewise, together with such geographical diversity, there is a cultural and ethnical mixture that needs to be considered, i.e. Quechua and Aymara populations are among the most common ethnic groups living in high altitude settings. Thus, it is hypothesized that well-known risk factors will play a different role depending on the setting and environmental factors surrounding individuals [14]. Therefore, this study aimed to estimate the incidence of T2D in four settings at different stages of urbanization and altitude in Peru. In addition, modifiable lifestyle behaviors as well as anthropometric markers were evaluated as potential risk factors, and their respective population attributable fractions (PAF) were estimated.

## 2 Methods

### 2.1 Study design and settings

**Fig. 1:**
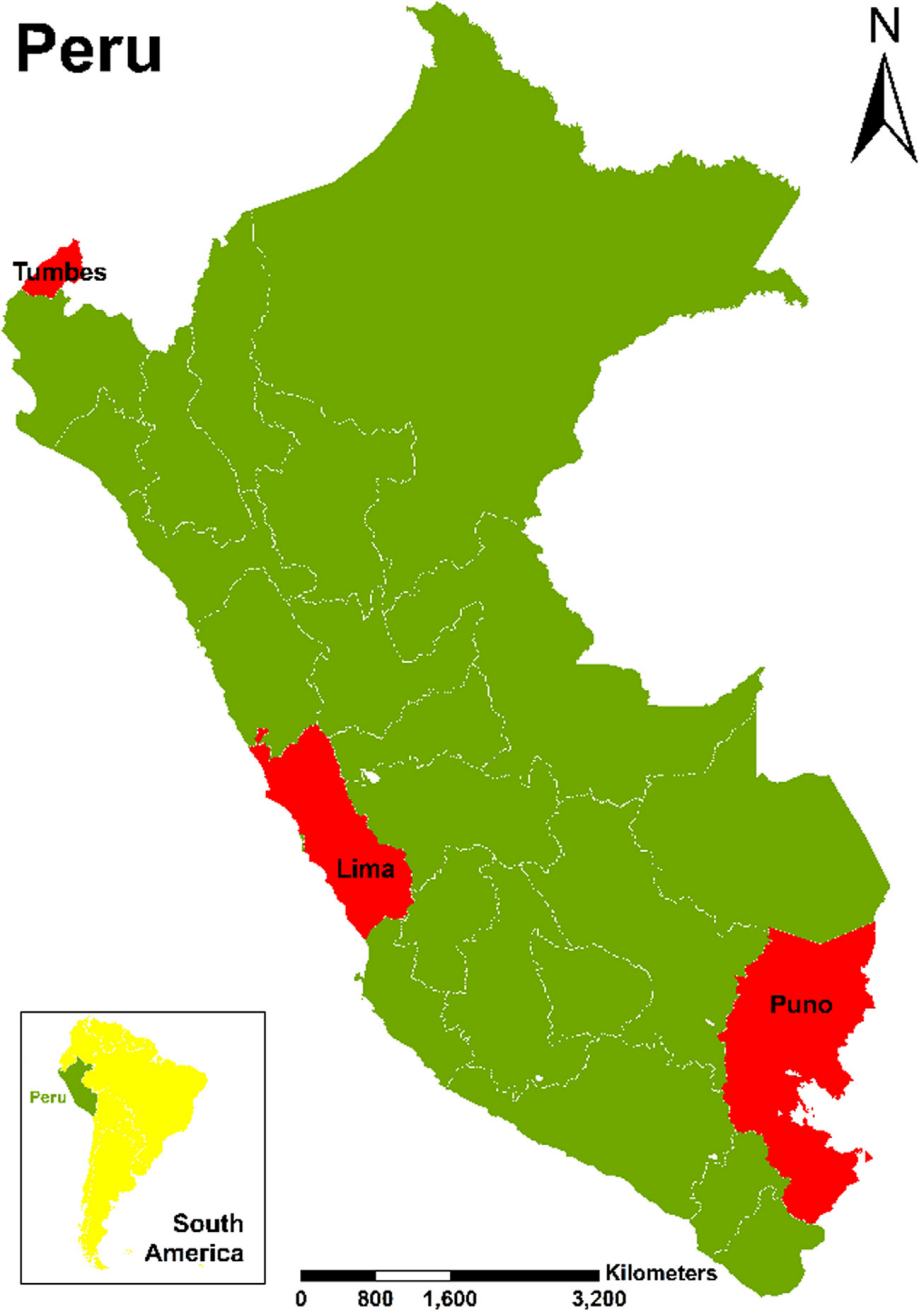
Map of Peru indicating the CRONICAS Cohort Study’s sites.

The CRONICAS Cohort Study was conducted in four Peruvian sites with varying degrees of altitude and urbanization (Fig. 1): Pampas de San Juan de Miraflores, in Lima, Latin America’s fourth largest city, a highly-urbanized area located at the sea level; Puno, located at 3825 meters above sea level, contributed one urban and one rural site; and Tumbes, a semiurban setting in the coastal North of Peru, also at sea level [15]. Enrollment started in September 2010 and follow-up was conducted, on average, 30 months after initial assessment. Rural and urban sites were defined as per Peru’s National Institute of Statistics and Informatics [16]: an urban area has ⩾100 homes grouped together, whereas a rural area has <100 households grouped together or if there are ⩾100 dispersed households.

### 2.2 Study population and sampling

Subjects were selected following a single-stage random sampling procedure. Individuals aged ⩾35 years who were permanent residents in the selected study sites were potentially eligible. At each site, a sex and age (35–44, 45–54, 55–64, and 65+ years) stratified random sample of potential participants was taken from the most updated census available. Only one subject per household was considered eligible and invited to the study. Pregnant women, bedridden individuals and those who were not able to provide consent were excluded. The study sample is not nationally representative, yet it is informative of the geographical diversity of study settings and other Peruvian population that share similar socioeconomic and geographic characteristics. Further details about the sampling procedures are described elsewhere [15].

**Fig. 2:**
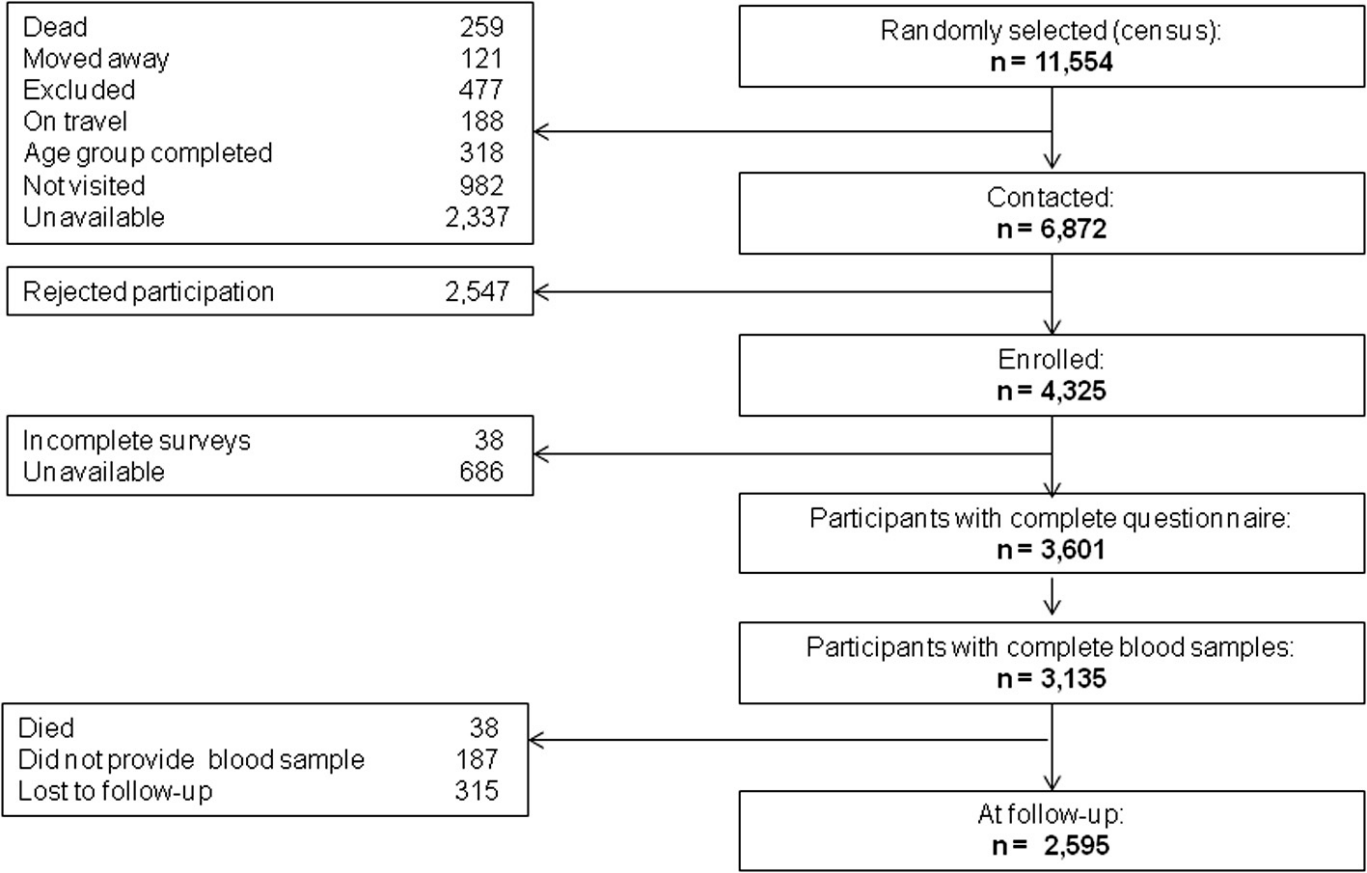
Baseline enrollment and follow-up of the CRONICAS Cohort Study.

At baseline, the cohort’s enrolment process was deemed complete when at least 1000 subjects in Lima and Tumbes sites were recruited together with 500 subjects from each of Puno’s urban and rural areas. The criterion for completion of recruitment was completion of questionnaires, blood laboratory tests, and clinical measurements. At baseline, 11,554 subjects were randomly selected, 6872 contacted, and 4325 enrolled. Once the baseline assessment was due, there were 3601 subjects with complete questionnaires, of whom 3232 had complete clinical measurement, and 3135 with complete blood samples (details in Fig. 2).

### 2.3 Definitions

The outcome of interest was T2D, defined as fasting blood glucose ⩾126 mg/dl (7.0 mmol/L) after a fasting period of 8–12 h, or current T2D pharmacological treatment prescribed by a physician [17]. In addition, for incidence calculations, a new case was considered if participant reported having newly started medication during follow-up and/or if blood glucose criteria were met. Whole blood (13.5 ml) was withdrawn from each participant, and fasting plasma glucose was measured using an enzymatic colorimetric method (GOD-PAP; Modular P-E/Roche-Cobas, Grenzach-Whylen, Germany).

Exposures of interest were divided in two groups: geographical-related variables and modifiable risk factors. Among the geographical variables, urbanization of the study site (highly urbanized Lima, urban Puno, rural Puno, and semi-urban Tumbes) and altitude (high altitude vs. sea level) were considered. Modifiable risk factors evaluated at baseline included: daily smoking of at least 1 cigarette per day, self-reported; hazardous drinking, based on the Alcohol Use Disorders Identification Test (⩾8 points in the score) [18]; number of hours of TV watching per day (<2, and ⩾2 h per day) [19]; transport-related physical inactivity, considering only the transport-related physical activity domain of the IPAQ and defined as not reporting walking or cycling trips in the last 7 days (i.e. a single walk or cycle trip for 10 min or more was considered to be classified as physical active) [20]; and fruits and vegetables intake, categorized according to the WHO recommendation (<5 and ⩾5 portions per day) [21].

Some anthropometric measurements mainly focused on weight and adiposity together with some laboratory markers were also assessed as potential risk factors: body mass index (BMI, <25, 25–29.9, and ⩾30 kg/m^2^); central obesity, based on waist circumference and categorized according to cut-off points for non-Caucasian population (<80 vs. ⩾80 cm for females, and <90 vs. ⩾90 cm for males); and metabolic syndrome [22]. In addition, hypertension, defined as systolic blood pressure ⩾140 mmHg or diastolic blood pressure ⩾90 mmHg or previous physician diagnosis and current pharmacological treatment [23], was also evaluated.

Other variables assessed at baseline and considered in the analysis were sex; age (35–44, 45–54, 55–64, and ⩾65 years); education (<7, 7–11, and ⩾12 years); socioeconomic status derived from assets possession and household facilities, and split in tertiles (low, middle, and high); and first language learnt at home (Spanish, Quechua or Aymara) as a proxy of ethnicity.

### 2.4 Procedures

Fieldwork activities and procedures of the CRONICAS Cohort Study have been described in detail elsewhere [15]. Briefly, at baseline, fieldworkers visited households to contact potential participants and include them in the study. The follow-up visit was conducted, on average, 30 months from baseline assessment. In both evaluations, subjects responded to a face-to-face questionnaire conducted by trained community health workers using paper-based formats. After completing the questionnaire, an appointment was made for a clinical assessment in order to guarantee an adequate fasting period. A total of 13.5 ml of blood was drawn at each clinical assessment. Standing height and waist circumference, in triplicate, was measured using standardized techniques. Weight was assessed using the TBF-300A body composition analyzer (TANITA Corporation, Tokyo, Japan). After a 5-min resting period, blood pressure was also measured in triplicate using an automatic monitor OMRON HEM-780 validated for adult population, and the average of the last two measurements were used in the analysis.

### 2.5 Biostatistical analysis

Analyses were conducted using STATA 13.0 (StataCorp, College Station. TX, USA). Population characteristics were tabulated according to the presence of T2D at baseline, and comparisons were performed using Chi-squared test or Fisher exact test accordingly. For longitudinal analyses, incidence rates per 100 person-years of follow-up and 95% confidence intervals (95%CI) were estimated excluding those who had diabetes at baseline. Incidence estimates were obtained according to predefined exposures of interest, i.e. geographical variables and modifiable risk factors.

Generalized linear models were used to determine the strength of association between T2D and exposures of interest, controlling for potential confounders. Overall and site-specific crude and adjusted models were also generated to determine relative risks (RR). Given the number of confounders and the potential correlation between them, the variance inflation factor was used to determine collinearity and exclude high correlated variables from the model if needed. Finally, population attributable fractions (PAF) were estimated by using the *punaf* command in STATA.

### 2.6 Ethics

Ethical approval was obtained from Institutional Review Boards at Universidad Peruana Cayetano Heredia and Asociación Benéfica PRISMA, in Peru, and the Johns Hopkins University, in the US. Participants provided verbal consent due to major illiteracy rates, especially in rural areas.

## 3 Results

A total of 3135 subjects were included in the analysis, with a mean age of 55.8 years (inter-quartile range: 45.3–65.0) and 48.5% were males. Characteristics of the study population according to T2D diagnosis at baseline are detailed in Table 1.

**Table 1:** Baseline population characteristics according to the presence of type 2 diabetes.

	Without diabetes	With diabetes	*p*-value
*Socio-demographics, n (%)*			
Sex	*n* = 2902	*n* = 221	0.17
Male	1425 (49.1%)	98 (44.3%)	
Age	*n* = 2902	*n* = 222	<0.001
<45 years	743 (25.6%)	17 (7.7%)	
45–54 years	743 (25.6%)	57 (25.7%)	
55–64 years	720 (24.8%)	73 (32.9%)	
65+ years	696 (24.0%)	75 (33.8%)	
Education level	*n* = 2902	*n* = 222	0.03
<7 years	1312 (45.2%)	120 (54.1%)	
7–11 years	963 (33.2%)	67 (30.2%)	
12+ years	627 (21.6%)	45 (15.8%)	
Socioeconomic status	*n* = 2904	*n* = 222	0.38
Lowest tertile	928 (32.0%)	61 (27.5%)	
Middle tertile	981 (33.8%)	79 (35.6%)	
Highest tertile	995 (34.3%)	82 (36.9%)	
Study setting	*n* = 2904	*n* = 222	<0.001
Lima	974 (33.5%)	59 (26.6%)	
Urban Puno	481 (16.6%)	39 (17.6%)	
Rural Puno	525 (18.1%)	17 (7.7%)	
Tumbes	924 (31.8%)	107 (48.2%)	
Setting altitude	*n* = 2904	*n* = 222	0.004
High altitude	1006 (34.6%)	56 (25.2%)	
First language learnt	*n* = 2903	*n* = 222	0.004
Spanish	1825 (62.9%)	160 (72.1%)	
Quechua	578 (19.9%)	42 (18.9%)	
Aymara	500 (17.2%)	20 (9.0%)	

*Lifestyles behaviors, n (%)*	*n* = 2904	*n* = 222	<0.001
Family history of diabetes	345 (11.9%)	75 (33.8%)	
	*n* = 2904	*n* = 222	0.84
Daily smoking⁎	95 (3.3%)	6 (2.7%)	
	*n* = 2904	*n* = 222	0.01
Hazardous drinking	408 (14.1%)	18 (8.1%)	
	*n* = 2902	*n* = 222	0.007
TV watching for 2+ hours per day	1222 (42.1%)	114 (51.4%)	
	*n* = 2904	*n* = 222	<0.001
Transport-related physical inactivity	278 (9.6%)	41 (18.4%)	
	*n* = 2902	*n* = 222	<0.001
Fruits and vegetables (5+ portion/day)	111 (3.8%)	21 (9.5%)	

*Measurements, n (%)*			
Body mass index	*n* = 2906	*n* = 222	<0.001
Normal (<25 kg/m^2^)	873 (30.0%)	42 (19.3%)	
Overweight (⩾25 and <30 kg/m^2^)	1279 (44.0%)	89 (40.8%)	
Obese (⩾30 kg/m^2^)	754 (26.0%)	87 (39.9%)	
Waist circumference	*n* = 2905	*n* = 218	<0.001
IDF central obesity	2077 (71.5%)	193 (88.5%)	
Hypertension	*n* = 2906	*n* = 218	<0.001
Measured or physician-diagnosed	693 (23.9%)	111 (50.9%)	
Metabolic syndrome	*n* = 2905	*n* = 217	<0.001
Yes	1273 (43.8%)	192 (88.5%)	

Results may not add due to missing values.

⁎ *P*-values were calculated using Fisher exact test instead of Chi-squared test.

### 3.1 Prevalence and incidence of diabetes

Overall prevalence of T2D at baseline was 7.1% (223/3140; 95%CI 6.3–8.1%) and there was a clear difference between study sites (*p* < 0.001). During the follow-up phase, 315 (10.0%) individuals were lost to follow-up, 187 (6.0%) did not provide a blood sample, and 38 (1.2%) died. Of the 2,595 re-contacted, 223 (8.6%) were further excluded from incidence calculations because of their diabetes status at baseline. Mean time of follow-up was 2.4 (SD: 0.4) years, accruing a total of 6207 person-years of follow-up, and 121 new cases of T2D were identified which translates into an overall incidence of 1.95 (95%CI 1.63–2.33) per 100 person-years. Further details of T2D incidence, crude RR and 95%CI, organized by characteristics of the study population are presented in Table 2.

**Table 2:** Incidence of type 2 diabetes according to population characteristics.

	Incidence (95%CI)	Bivariable model
	per 100 person-years	RR (95%CI)
*Socio-demographics*		
Sex		
Female	1.97 (1.54–2.52)	1 (reference)
Male	1.93 (1.49–2.50)	0.96 (0.68–1.36)
Age		
35–44 years	1.24 (0.80–1.93)	1 (reference)
45–54 years	1.92 (1.35–2.73)	1.55 (0.89–2.68)
55–64 years	2.92 (2.18–3.89)	2.30 (1.37–3.84)
65+ years	1.71 (1.14–2.55)	1.45 (0.81–2.60)
Education level		
<7 years	1.82 (1.38–2.41)	1 (reference)
7–11 years	1.64 (1.17–2.30)	0.83 (0.54–1.27)
12+ years	2.14 (1.49–3.08)	1.08 (0.69–1.69)
Socioeconomic status		
Lowest tertile	1.66 (1.16–2.38)	1 (reference)
Middle tertile	1.72 (1.205–2.38)	0.96 (0.60–1.54)
Highest tertile	2.39 (1.83–3.12)	1.29 (0.84–2.00)
First language learnt		
Spanish	2.00 (1.61–2.47)	1
Quechua	2.26 (1.55–3.29)	1.17 (0.77–1.78)
Aymara	1.25 (0.67–2.32)	0.78 (0.41–1.48)

*Lifestyles behaviors*		
Family history of diabetes		
No	1.81 (1.48–2.21)	1 (reference)
Yes	2.91 (1.93–4.38)	1.55 (1.01–2.41)
Daily smoking		
No	1.92 (1.60–2.31)	1 (reference)
Yes	2.70 (1.21–6.01)	1.34 (0.61–2.94)
Hazardous drinking		
No	1.91 (1.58–2.32)	1 (reference)
Yes	2.18 (1.38–3.47)	1.13 (0.70–1.84)
TV watching		
<2 h per day	1.85 (1.45–2.36)	1 (reference)
2+ hours per day	2.08 (1.60–2.70)	1.08 (0.76–1.53)
Transport-related physical inactivity		
No	1.83 (1.51–2.23)	1 (reference)
Yes	2.76 (1.72–4.45)	1.51 (0.92–2.48)
Fruits and vegetables consumption		
<5 portions per day	1.93 (1.60–2.31)	1 (reference)
5+ portions per day	2.57 (1.15–5.71)	1.28 (0.58–2.84)

*Measurements*		
Body mass index		
Normal (<25 kg/m^2^)	0.87 (0.53–1.45)	1 (reference)
Overweight (⩾25 and <30 kg/m^2^)	1.68 (1.26–2.24)	1.81 (1.02–3.21)
Obese (⩾30 kg/m^2^)	3.48 (2.70–4.49)	3.72 (2.13–6.50)
Waist circumference (IDF)		
No central obesity	0.80 (0.47–1.39)	1 (reference)
Central obesity	2.35 (1.95–2.84)	2.79 (1.58–4.92)
Hypertension		
No	1.76 (1.42–2.19)	1 (reference)
Yes	2.54 (1.85–3.49)	1.43 (0.98–2.07)
Metabolic syndrome		
No	0.89 (0.62–1.28)	1 (reference)
Yes	3.16 (2.57–3.90)	3.42 (2.28–5.13)

### 3.2 Study site and modifiable factors as risk factors for diabetes

A gradient towards higher incidence rate of T2D according to urbanization was observable: there were 1.52 new T2D cases per 100 person-years in rural Puno, while this estimate was 1.87, 1.96, and 2.50 for semi-urban Tumbes, Lima, and urban Puno, respectively. However, after controlling for several confounders, there was no evidence of the impact of urbanization on the incidence of T2D. Nonetheless, subjects living in high altitude sites had a 58% higher risk of developing T2D (RR = 1.58; 95%CI 1.01–2.48) when compared to those living at sea level (Table 3).

**Table 3:** Association between study site characteristics and the risk of diabetes: crude and adjusted models.

	Incidence (95%CI)	Crude model	Adjusted model^a^	Adjusted model^b^
	per 100 person-years	RR (95%CI)	RR (95%CI)	RR (95%CI)
*Study setting*				
Lima	1.96 (1.46–2.62)	1 (reference)	1 (reference)	1 (reference)
Urban Puno	2.50 (1.66–3.76)	1.19 (0.73–1.93)	1.32 (0.75–2.30)	1.45 (0.84–2.48)
Rural Puno	1.52 (0.86–2.68)	0.71 (0.38–1.33)	1.13 (0.56–2.29)	1.52 (0.73–3.18)
Tumbes	1.87 (1.37–2.53)	0.96 (0.64–1.45)	1.02 (0.66–1.58)	0.86 (0.54–1.36)

*Setting altitude*				
Low	1.91 (1.55–2.36)	1 (reference)	1 (reference)	1 (reference)
High	2.05 (1.47–2.85)	1.20 (0.82–1.75)	1.23 (0.79–1.91)	1.58 (1.01–2.48)

a The model was adjusted by sex, age, education level, and socioeconomic status.

b Model adjusted for sex, age, education level, socioeconomic status, family history of diabetes, daily smoking, hazardous drinking, TV watching for 2+ hours per day, transport-related physical inactivity, fruits and vegetables consumption, body mass index, and metabolic syndrome.

None of the modifiable risk factors studied were associated with an increased risk of T2D. However, variables related to obesity, i.e. BMI, waist circumference, and metabolic syndrome, were associated with increased risk of developing T2D. PAF of all obesity-related variables were over 20% (Table 4). Site-specific analysis showed that transport-related physical inactivity was the only factor that increased T2D risk in the highly urbanized setting (RR = 2.96), whereas TV watching for 2+ hours per day (RR = 1.56) and transport-related physical inactivity (RR = 12.7) were associated with greater risk of T2D in rural Puno (online supplement: E-Table 1). Based on altitude, only transport-related physical inactivity was associated with T2D (RR = 1.68) in sea level settings (online supplement: E-Table 2).

**Table 4:** Modifiable factors and the risk of type 2 diabetes: adjusted models and population attributable fractions (PAF).

	Adjusted model^a^	Population attributable fraction
	RR (95%CI)	
*Lifestyles behaviors*		
Daily smoking	1.35 (0.61–3.01)	1.3%
Hazardous drinking	1.24 (0.74–2.09)	2.9%
TV watching for 2+ hours per day	1.12 (0.78–1.61)	5.0%
Transport-related physical inactivity	1.67 (0.98–2.84)	5.7%
Fruits and vegetables: 5+ portions/day	1.24 (0.56–2.74)	1.0%

*Measurements*		
Body mass index (vs. normal)		
Overweight (⩾25 and <30 kg/m^2^)	1.89 (1.04–3.43)	36.5%
Obese (⩾30 kg/m^2^)	3.99 (2.23–7.14)	54.9%
Central obesity (IDF)	2.88 (1.63–5.09)	58.3%
Hypertension	1.35 (0.91–2.01)	8.2%
Metabolic syndrome	3.34 (2.23–5.02)	52.4%

Population attributable fractions were calculated using the *punaf* command in STATA.

a The model was adjusted by sex, age, education level, socioeconomic status, and study setting.

Obesity at baseline had the highest attributable risk for the developing T2D, although results varied markedly by setting: PAF of obesity based on BMI was 59.2% in highly urbanized Lima, 13.9% in urban Puno, 32.4% in rural Puno, and 79.8%, in semi-urban Tumbes. Moreover, according to altitude, PAFs of obesity was 69.8% in sea level areas, whereas it was 26.5% in high altitude settings.

## 4 Discussion

### 4.1 Main findings

Although there was no evidence of difference in the incidence of T2D according to the degree of urbanization as depicted by study sites; unadjusted results revealed an interesting pattern: T2D incidence rate increased from rural Puno throughout semi-urban Tumbes, Lima and urban Puno. On the other hand, individuals from high altitude sites were found to have a higher risk of developing T2D. Obesity, measured as BMI and waist circumference, was the factor with higher attributable risk for T2D development, but PAF varied substantially along with urbanization and altitude. The contribution of the other modifiable risk factors in diabetes development also differed by geographical characteristics of study sites. This information affords understanding of diabetes-related disease burden in LMIC by providing information, derived from longitudinal studies, to identify T2D hotspots for resource prioritization.

### 4.2 High altitude as a potential risk factor for diabetes

Similar to our baseline results, previous studies have reported a lower prevalence of T2D in high altitude compared to sea level settings [11], [12]; however, longitudinal evidence of progression towards T2D in such settings is nearly absent. Our study expands on such previous studies by demonstrating, using a prospective design, that individuals living at high altitude areas (3825 meters above sea level) are at greater risk of incident diabetes after controlling for sociodemographic, lifestyle behaviors, and anthropometric measurements.

Information regarding the impact of chronic exposure to high altitude on developing diabetes, however, is limited. The Atherosclerosis Risk in Communities (ARIC) cohort study reported that lower forced vital capacity (FVC) was an independent predictor of incident type 2 diabetes [24]; however, high altitude in our context has been associated with larger lung capacity, but lower forced expiratory volume – forced vital capacity (FEV/FVC) ratio [25]. A previous report from our group, using a subsample of participants from Puno found that, after controlling for several confounders, lower oxyhemoglobin saturation was associated with cardiometabolic factors such as metabolic syndrome and higher waist circumference [26]. There is some evidence that acute exposure to high altitude settings, through hypoxia regulated pathways, may increase insulin sensitivity [27]. However, other studies in low-oxygen environments have found that, potentially through systemic inflection [28] and sympathetic nervous system activation [29], hypoxemia can disrupt glycemic control.

Alternatively, high altitude in our study may imply differences related to ethnicity given rural and urban Puno are primarily Quechua and Aymara populations. As previous studies have reported at least similar prevalence rates of diabetes in high altitude aboriginal groups [30], [31], and obesity markers have lower population attributable fractions among individuals from high altitude settings, alternative explanations might include changes in lifestyles, mainly characterized by high consumption of saturated fat and refined sugars [32], variables not available in our analysis. Genetic susceptibility should also be considered; however, the genetic admixture in Peruvians is very high [33], with many groups sharing common Native American ancestry and thus requiring a deeper study to understand its contribution to the observed differences in the incidence of diabetes.

Future studies are needed to understand the pathophysiological mechanisms of T2D progression in high altitude. Among the alternative explanations, we ought to consider the role of fetal programming, low birth weight, neonatal determinants, and childhood chronic undernutrition on the adult onset of diabetes and other metabolic traits. In addition, growth variability in early life may play a contributing role. Some studies have reported an association between low birth weight, or low weight at one year, with the risk of diabetes in adult life [34]. Other studies have linked shorter adult stature [35], or shorter relative leg length [36], with adult diabetes risk. Relative leg length is not correlated with birth weight, and acts as a marker of the quality of post-natal growth [37]. The mechanism underlying these associations has been identified as nutritional impairment of pancreatic beta cells during early development [38]. Of particular relevance here, we have previously shown that children born at high altitude in Peru have relatively shorter legs compared to those born at low altitude [39]. If, as in other studies, these contrasting growth patterns mark variability in pancreatic beta cell development, then the poorer growth of high-altitude children would be predicted to increase their diabetes risk for any given level of obesity.

### 4.3 Obesity and other risk factors for incident diabetes

Obesity, assessed as BMI, waist circumference, and partially by metabolic syndrome, was the risk factor with the highest attributable risks for incident T2D across sites in our study. Both being overweight and obese have been associated with increased risk of diabetes, cancer, cardiovascular diseases, among other comorbidities [40]. Based on the PAF, central obesity was the most important risk factor explaining the burden of diabetes in our population. Central obesity shows PAF values ranging from 26% in urban Puno to 86% in semiurban Tumbes, almost similar to the variation of BMI. A previous meta-analysis concluded that waist circumference was a modestly stronger predictor of diabetes than body mass index, although this difference was not significant [41]. Thus, as in other countries, the pandemic of obesity, and hence cardiovascular disease and T2D, is reaching even the most poorer and remote areas of LMIC. For example, TV watching and transport-related physical inactivity, indicators of sedentarism, and related to obesity, were strongly associated with incident diabetes in rural Puno, pointing out that even rural areas are currently being affected by unhealthy lifestyle behaviors.

### 4.4 Public health implications

Over the course of 2.5 years, we found a major difference in expected patterns of incidence of T2D by study site, a pattern that would have gone unnoticed if we were to rely solely on prevalence or disease burden type of investigations. As about 140 million people worldwide live at high altitudes, i.e. >2500 meters above sea level [13], our findings suggest a signal towards a prioritization of high altitude areas in diabetes prevention efforts to avoid the permanent installation of T2D and metabolic damage in low-prevalence settings.

Our results also suggest that effective strategies are needed to reduce the burden of diabetes in our populations. Obesity should be the center of these interventions, which would decrease dramatically the emergence of new cases of diabetes. These interventions should be cognizant of the different magnitude of effect of obesity on diabetes incidence by geographical settings [4], which could introduce pragmatic elements to design adequate interventions with sufficient intensity. Our results regarding population attributable fractions are reassuring: if obesity, a modifiable risk factor, were not to be present, the proportion of diabetes that could be reduced would be at least half of cases of diabetes, and such impact could be higher depending on the characteristics of the study site. Yet, despite the availability of evidence of effective interventions that reduce the risk of developing diabetes involving healthier diets and exercise, as well as pharmacological treatment [2], the obesity pandemic has dramatically accelerated in the last decades. Much of the information regarding the rise of obesity, especially in LMIC, is derived from estimates aggregated at the national level, yet our findings call for close monitoring of obesity trends within country and beyond country-level aggregates, especially for rural populations as well as those living at high altitude settings.

### 4.5 Strengths and limitations

To our knowledge, this is the first longitudinal study assessing the progression of diabetes according to geographical variation and altitude. Our estimates were calculated using a population-based study in settings with different geographical characteristics. However, some limitations must be noted. First, T2D was defined using fasting blood glucose and current newly started treatment instead of the gold standard, i.e. oral glucose tolerance test; as a result, some cases of diabetes may have been missed. Nevertheless, our definitions and results are similar to those used in existing reports. Second, some selection bias might affect our results. Despite the random selection of participants at baseline, we selected specific study sites which might not be representative of all Peruvian settings. Moreover, the rejection rate at baseline was not trivial (see details in Fig. 2). In addition, as other longitudinal studies, rejection to participate during follow-up may have biased our estimates. Third, results could also reflect the effect of unmeasured confounders such as diet patterns. We included fruits and vegetables intake but we did not assess fat intake, refined-sugar consumption, among other dietary products. At some extent, this limitation might have been overcome by including sex and socioeconomic status in the regression model. A study in Mexico reported different levels of fat, saturated fat, and cholesterol between men and women and among low, middle and high socioeconomic level [42]. Third, power might be an issue, mainly due to short term follow-up, i.e. 30 months on average, as many recognized risk factors for diabetes were not significant in our results. However, as PAFs assess the contribution of a risk factor to a disease, they can provide a better understanding of the role of these factors in the involved populations. Fourth, over-adjustment could be an issue as our models included several variables implicit with urbanization, i.e. education, socioeconomic status, TV watching, physical inactivity, etc. However, our models involved variables that are part of the usual assessment of traditional risk factors. In addition, two different models, partially and full adjusted, have been provided to explore the possibility of over-adjustment.

## 5 Conclusions

Geographical variation, mainly high altitude, may play a role in the risk of developing T2D and the pathophysiology leading to this relationship needs to be further investigated and understood. Moreover, impact of risk factors on incident diabetes cases varied substantially according to geographical characteristics, and new cases of diabetes can be largely attributable to obesity. These findings can well support the design of appropriate context-specific disease intervention strategies aimed to reduce the rising burden of diabetes.

## Funding

This project has been funded in whole with Federal funds from the United States National Heart, Lung, and Blood Institute, 10.13039/100000002National Institutes of Health, Department of Health and Human Services, under Contract No. HHSN268200900033C. William Checkley was further supported by a Pathway to Independence Award (R00HL096955) from the National Heart, Lung and Blood Institute. Liam Smeeth is a Senior Clinical Fellow and Antonio Bernabe-Ortiz is a Research Training Fellow in Public Health and Tropical Medicine (103994/Z/14/Z), both funded by 10.13039/100004440Wellcome Trust.

## Contributors

AB-O, RMC-L and JJM conceived the idea of the manuscript. AB-O drafted the first version of the manuscript and led the statistical analysis with support of RMC-L. JJM, RHG, WC and LS conceived, designed and supervised the overall study. JJM, AB-O and WC coordinated and supervised fieldwork activities in Lima, Tumbes, and Puno study sites. All authors participated in writing of manuscript, provided important intellectual content and gave their final approval of the version submitted for publication.

## Conflict of interest

The authors have nothing to disclose.
