# Supplementary material for: Geographical variation in the progression of type 2 diabetes in Peru: The CRONICAS Cohort Study
Source: Diabetes Res Clin Pract. 2016 Nov;121:135–45. doi: 10.1016/j.diabres.2016.09.007 (PMC5154928; doi:10.1016/j.diabres.2016.09.007)
Supplement: Supplementary data 1 [file mmc1.doc]

# ONLINE SUPPLEMENT

## E-Table 1: Modifiable factors and the risk of diabetes according to study site: adjusted models and population attributable fractions (PAF)

|  | **Lima** | | **Urban Puno** | | **Rural Puno** | | **Tumbes** | |
| --- | --- | --- | --- | --- | --- | --- | --- | --- |
|  | **RR*** | **PAF** | **RR*** | **PAF** | **RR*** | **PAF** | **RR*** | **PAF** |
| **Lifestyles behaviors** |  |  |  |  |  |  |  |  |
| *Daily smoking* | 1.37 | 1.2% | -- | -- | -- | -- | 1.84 | 4.4% |
| *Hazardous drinking* | 1.32 | 3.2% | 1.47 | 9.7% | -- | -- | 1.22 | 1.7% |
| *TV watching for 2+ hours per day* | 1.12 | 5.3% | 0.97 | -1.3% | **1.56** | 9.0% | 1.08 | 3.7% |
| *Transport-related physical inactivity* | **2.96** | 10.3% | -- | -- | **12.7** | 8.4% | 1.21 | 3.8% |
| *Fruits and vegetables: 5+ portions/day* | 2.00 | 5.6% | 0.62 | -2.7% | -- | -- | -- | -- |
| **Measurements** |  |  |  |  |  |  |  |  |
| *Body mass index (vs. normal)* |  |  |  |  |  |  |  |  |
| Overweight (≥25 and <30 kg/m^2^) | 1.74 | 36.6% | 0.75 | -22.0% | 2.20 | 32.2% | **4.61** | 6.7% |
| Obese (≥30 kg/m^2^) | **4.53** | 59.2% | 1.44 | 13.9% | **5.91** | 32.4% | **8.44** | 79.8% |
| *Central obesity (IDF)* | **2.94** | 60.1% | 1.51 | 26.4% | **3.90** | 55.8% | **8.10** | 85.5% |
| *Hypertension* | 1.69 | 15.4% | 1.28 | 5.6% | 1.39 | 4.6% | 1.13 | 3.7% |
| *Metabolic syndrome* | **2.69** | 46.0% | 1.52 | 18.6% | **4.70** | 50.1% | **8.25** | 79.3% |

(--) = not calculable. Bold estimates are significant (p < 0.05).

* Model adjusted for sex, age, education level, and socioeconomic status.

## E-Table 2: Modifiable factors and the risk of diabetes according to setting altitude: adjusted models and population attributable fractions (PAF)

|  | **Low altitude** | | **High altitude** | |
| --- | --- | --- | --- | --- |
|  | **RR*** | **PAF** | **RR*** | **PAF** |
| **Lifestyles behaviors** |  |  |  |  |
| *Daily smoking* | 1.65 | 2.7% | -- | -- |
| *Hazardous drinking* | 1.30 | 2.7% | 1.01 | 0.2% |
| *TV watching for 2+ hours per day* | 1.09 | 4.2% | 1.14 | 4.9% |
| *Transport-related physical inactivity* | **1.68** | 7.5% | 1.86 | 1.4% |
| *Fruits and vegetables: 5+ portions/day* | 1.48 | 1.9% | 0.56 | -2.2% |
| **Measurements** |  |  |  |  |
| *Body mass index (vs. normal)* |  |  |  |  |
| Overweight (≥25 and <30 kg/m^2^) | **2.73** | 17.4% | 1.24 | 9.0% |
| Obese (≥30 kg/m^2^) | **5.80** | 69.8% | 2.38 | 26.5% |
| *Central obesity (IDF)* | **3.99** | 70.6% | 2.15 | 41.3% |
| *Hypertension* | 1.37 | 9.3% | 1.26 | 4.8% |
| *Metabolic syndrome* | **4.05** | 61.3% | **2.28** | 32.3% |

(--) = not calculable. Bold estimates are significant (p < 0.05).

* Model adjusted for sex, age, education level, and socioeconomic status.
